# Risk factors associated with tongue lesions: a propensity score-matched case-control study

**DOI:** 10.4317/medoral.24836

**Published:** 2021-12-07

**Authors:** Laura González-Álvarez, María José García-Pola

**Affiliations:** 1 Assistant Professor. Department of Surgery and Medical-Surgical Specialties, Faculty of Medicine and Sciences of the Health. Oviedo University, Spain; 2 Professor of Oral Medicine. Department of Surgery and Medical-Surgical Specialties, Faculty of Medicine and Sciences of the Health. Oviedo University, Spain

## Abstract

**Background:**

to analyse the potential risk factors of tongue lesions, comparing the results with a control group.

**Material and Methods:**

An observational, case-control study was designed. The study included a case group comprising 336 patients with tongue lesions and 336 sex and age-matched controls. We recorded tobacco/alcohol habits, presence of dentures, allergies, medical conditions, and medications. Statistical analysis was performed via logistic regression models to estimate the odds ratio (OR) adjusted for gender, age, tobacco, and alcohol using propensity score-matching analysis (PSM).

**Results:**

According to the final PSM model, patients with tongue lesions were more likely to suffer from allergies (OR 2.13; 1.27-3.66) or medical conditions (OR 2.14; 1.19-3.85), and more likely to take medication (OR 1.99; 1.11-3.57). Elderly individuals were more prone to hairy tongue (OR 3.82; 1.53-10.47). Smoking was associated with coated tongue (OR 2.05; 1.12-3.63), hairy tongue (OR 3.77; 1.52-9.22) and median rhomboid glossitis (OR 40.49; 5.84-860.43). Allergic individuals were more likely to exhibit sublingual varices (OR 1.73; 1.02-2.88). Medical conditions increased the chances of having coated tongue (OR 2.44; 1.36-4.64) or crenated tongue (OR 2.70; 1.42-5.30). Arterial hypertension was associated with median rhomboid glossitis (OR 5.85; 1.08-34.18). Individuals on medication showed a higher risk of fissured tongue (OR 1.87; 1.20-2.94) and varices (OR 2.42; 1.58-3.80). Agents acting on the alimentary tract and metabolism increased the probability of fissured tongue (OR 2.31; 1.42-3.79).

**Conclusions:**

As far as we are aware, this is the first study on lingual pathology to include a PSM analysis. The results suggest that a history of allergies, the presence of medical conditions, and the use of medication are associated with increased probability of tongue lesions. The analysis of diseases and medications by subgroups requires studies matched by habits with larger sample sizes, in order to corroborate our observations.

** Key words:**Tongue lesions, fissured tongue, allergies, medications, risk factors, systemic diseases.

## Introduction

The prevalence of tongue lesions ranges from 2.39% to 15.1% in the general population (1,2). This variability may be due to differences in sample size and selection. In studies performed in university dental school clinics these lesions are observed in up to 52.2% of dental outpatients (3).

Interest in tongue lesions is due to it often being part of oral mucosal pathology, as well as its possible association with systemic diseases and medication. The most commonly documented lesions are geographic tongue, fissured tongue, coated tongue, hairy tongue, crenated tongue, sublingual varices, and median rhomboid glossitis.

Many studies have shown that the frequency of fissured tongue (2,4,5), sublingual varices (2,6-8) and hairy tongue (2,3,9) increases with age. Hairy tongue is also more common in men (3,9-11). Tobacco use has been related to coated tongue (3,11) hairy tongue (2-4,10), fissured tongue (3), and sublingual varices (7,8). In contrast, crenated (3), geographic (3,12-14) and the aforementioned fissured tongue have demonstrated inverse associations with tobacco use (4).

Regarding systemic diseases, studies performed in children have reported a correlation between geographic tongue and chronic diseases (15), as well as between fissured tongue and a history of allergy (16). In addition, studies performed in older people, or with broader age ranges, have reported a higher frequency of cardiovascular diseases (7,17), including arterial hypertension (6,8,18), in patients with sublingual varices. Arterial hypertension has also been associated with geographic tongue (12,19).

Some authors have noted the influence of medication in tongue lesions. Patients with sublingual varices (17), fissured tongue or geographic tongue have been shown to have a higher intake of cardiovascular agents (12,19). In addition, antibiotics (18) and topical steroids (12) have been related to fissured tongue, whereas systemic steroids have been associated with geographic tongue (14).

Findings related to the influence of diseases and medications on lingual pathology are scarce, and sometimes the results are contradictory or difficult to interpret. Bhattacharya *et al*. and Kaplan & Moskona did not find an association between diseases and tongue lesions (9,20). Shulman & Carpenter, and Miloğlu *et al*. also failed to establish a relationship between geographic tongue and diabetes or other conditions (13,14). However, in the study by Koay *et al*. the presence of diabetes was significantly higher in fissured tongue patients, and that relationship seemed to be unrelated to medication, such as oral hypoglycaemic agents (4).

Considering these contradictions, the aim of the present study is to evaluate the factors associated with tongue lesions, and to analyse the effect of medical conditions and medication, comparing the results with a control group.

## Material and Methods

- Study design and setting

The present observational case-control study was designed following the STROBE guidelines (appendix 1) (21). The participants were 672 consecutive patients who were referred at the Oral Medicine Section, of the Dental Clinics of the University of Oviedo, between January 2017 and July 2020.

The study was approved by the Ethics Committee of Principado de Asturias (nº 310/19), and all participants signed an informed consent, in accordance with data protection regulation and the Declaration of Helsinki.

- Patient selection

The case group was made up of 336 patients diagnosed with tongue lesions. The inclusion criteria were the clinical diagnosis of any of the following lesions: fissured tongue, geographic tongue, coated tongue, hairy tongue, crenated tongue, median rhomboid glossitis (MRG) and sublingual varices. The exclusion criteria were: 1) patients affected by congenital syndromes of the head and neck region; 2) pregnancy; 3) patients undergoing chemotherapy and/or radiotherapy, and 4) patients treated for an acute disease in the previous month.

The control group was made up of 336 patients that were seen in the same department for another type of benign pathology or for an oral health examination. The controls were matched for age and sex, and the same exclusion criteria as the case group were applied.

- Variables and data sources

The following variables were collected using an ad hoc questionnaire: demographic data (gender and age); harmful habits (tobacco and alcohol), use of dentures, history of allergies, medical conditions, and medication. The following groups of pathologies were recorded: endocrine, cardiovascular, respiratory and rheumatological. We also recorded specific diseases such as thyroid disorders, diabetes mellitus, arterial hypertension, cardiac insufficiency, asthma, and anxiety/depression.

Medications were categorised according to the first and second level of the Anatomical Therapeutic Chemical (ATC) classification system. We included the following groups from the first ATC level: A (Alimentary tract and metabolism), B (Blood and blood forming organs), C (Cardiovascular system), H (Systemic hormonal preparations, excluding sex hormones and insulins), M (Musculo-skeletal system), N (Nervous system), and R (Respiratory system). The most common first level groups (A, C, and N) were sub-divided into their most common second level subgroups: drugs for acid related disorders (ATC A02), drugs used in diabetes (ATC A10), agents acting on the renin–angiotensin system (ATC C09), lipid modifying agents (ATC C10), psycholeptics (ATC N05) and psychoanaleptics (ATC N06).

- Oral examination

All subjects were examined by the two authors simultaneously, with a strong concordance (kappa = 1). First, the medical history questionnaire was completed. Then, the examination was performed under artificial light in a dental chair, using a dental mirror, gauze, and saliva ejectors. The presence of fissured tongue was recorded according to the criteria from Feil & Filippi (22). Sublingual varices were diagnosed as described by Al-Shayyab & Baqain (7). Diagnosis of the remaining tongue lesions was based on the descriptions from Avcu & Kanli (3). During the oral examination, the presence of dentures was recorded.

- Statistical analysis

Initially, we performed a descriptive study of all of the variables. For the analytical study, variables were considered dichotomously (presence/absence, or yes/no). We divided the age variable into two categories, selecting the cut-off point at 60 years old. The relationships between qualitative variables were assessed by Pearson's chi-square test or Fisher's test. We performed logistic regression models to study the factors associated with the occurrence of tongue lesions. The multivariate model was built using stepwise selection with variables whose *p* value was <0.20 in the univariate analysis. To avoid a collinearity effect, the disease groups and the second ATC level were not included in the multivariate model. Additionally, we performed a propensity score-matching (PSM) analysis adjusted to gender, age, tobacco use, and alcohol use to reduce bias. Through the application of PSM, the participants of the case and control groups were paired for the variables mentioned above. This statistical method provided a balanced distribution of the selected characteristics in both groups, reducing the probability of introducing confounding factors. We used the R program (R Development Core Team), version 3.6.0. for the statistical analyses. Results were considered statistically significant when *p* <0.05.

## Results

- Participants and descriptive data

Both the case and control groups were made up of 235 women (69.9%) and 101 men (30.6%). The most frequently found lesion was fissured tongue (n=189; 56.2%) followed by sublingual varices (n=162; 48.21%), coated tongue (n=75; 22.3%), crenated tongue (n=68; 20.2%), geographic tongue (n=36; 10.7%), hairy tongue (n=27; 8.4%) and median rhomboid glossitis (n=10; 2.98%). At the time of diagnosis, 112 subjects had two tongue lesions and the mean number was 1.68.

- Statistical analysis

The distribution of demographic characteristics, harmful habits, and patients’ medical histories are summarised in Table 1. There were no significant differences in terms of age groups (*p*=0.699). In the case group there was a higher frequency of smoking (*p*=0.022), alcohol consumption (*p*<0.001), and history of allergies (*p*<0.001). In contrast, in the control group there were more denture wearers, but this difference was not statistically significant (*p*=0.071).

Patients with tongue lesions showed a higher frequency of medical conditions (*p*<0.001) than the control group. However, there were no significant differences in the presence of endocrine pathology (*p*=0.05), anxiety/depression (*p*=0.164), diabetes (*p*=0.145) or asthma (*p*=0.14).

In the sample, the number of medications taken ranged between 1 and 15 per day. Patients with tongue lesions had a higher intake of medications than the controls, with a mean of 2.03 vs 0.96 (*p*<0.001). Moreover, the medication intake in each category analysed was significantly higher in patients with tongue lesions, with the exception of ATC H (systemic hormonal preparations, excluding sex hormones and insulin) (*p*=0.056) and ATC A10 (drugs used in diabetes) (*p*=0.099).

The distribution of variables according to each tongue lesion is listed in Table 2. Table 3 shows the characteristics of the study population after PSM. The results of the logistic regression and PSM analyses are shown in Table 4. The presence of tongue lesions was significantly associated with allergy (OR 2.13; 95% CI 1.27-3.66; *p*=0.005), medical conditions (OR 2.14; 95% CI 1.19-3.85; *p*=0.011), and medication (OR 1.99; 95% CI 1.11-3.57; *p*=0.021).

Hairy tongue was more frequent in patients ≥60 years old (OR 3.82; 95% CI 1.53-10.47; *p*=0.005) and smokers (OR 3.77; 95% CI 1.52-9.22; *p*=0.004). Coated tongue and median rhomboid glossitis were also associated with smoking (OR 2.05; 95% CI 1.12-3.63; *p*=0.016 and OR 40.49; 95% CI 5.84-860.43; *p*=0.002, respectively). In patients with sublingual varices, the presence of allergies was more prevalent (OR 1.73; 95% CI 1.02-2.88; *p*=0.037).

Significant correlations were found between crenated or coated tongue and medical conditions (OR 2.70; 95% CI 1.42-5.30; *p*=0.003 and OR 2.44; 95% CI 1.36-4.64; *p*=0.004, respectively), and between median rhomboid glossitis and hypertension (OR 5.85; 95% CI 1.08-34.18; *p*=0.038).

Fissured tongue and sublingual varices were associated with medication (OR 1.87; 95% CI 1.20-2.94; *p*=0.006 and OR 2.42; 95% CI 1.58-3.80; *p*<0.001, respectively). Fissured tongue was also related to the consumption of agents acting on the alimentary tract and metabolism (OR 2.31; 95% CI 1.42-3.79; *p*=0.001). Geographic tongue was not associated with any of the factors we analysed.

**Table 1:**
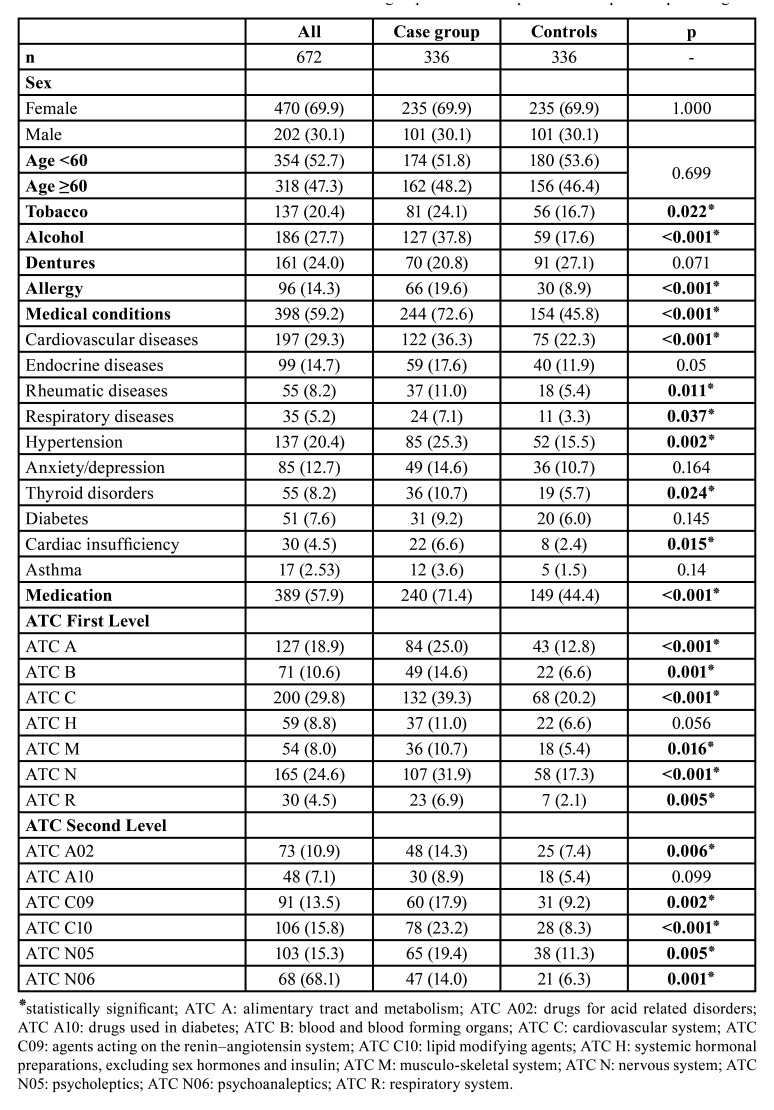
Distribution of variables in the case and control groups. Numbers in parentheses represents percentages.

**Table 2:**
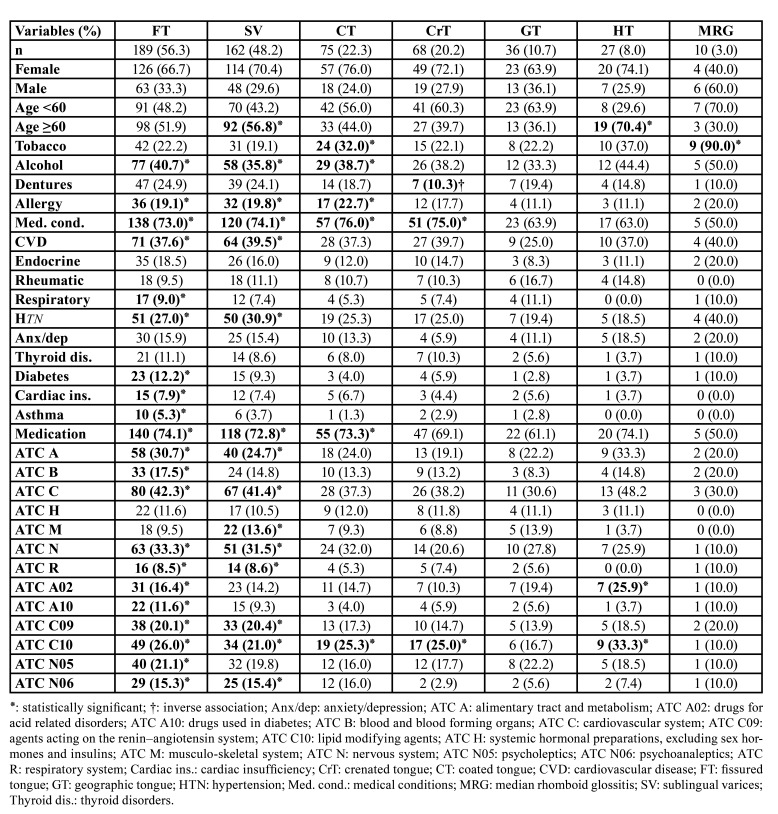
Distribution of variables according to tongue lesions. Numbers in parentheses represents percentages.

**Table 3:**
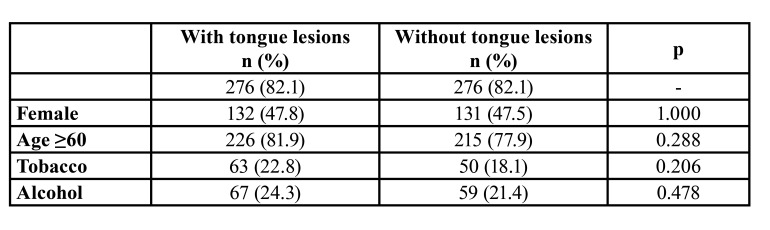
Characteristics of the study population after propensity score-matching (PSM). Numbers in parentheses represents percentages.

**Table 4:**
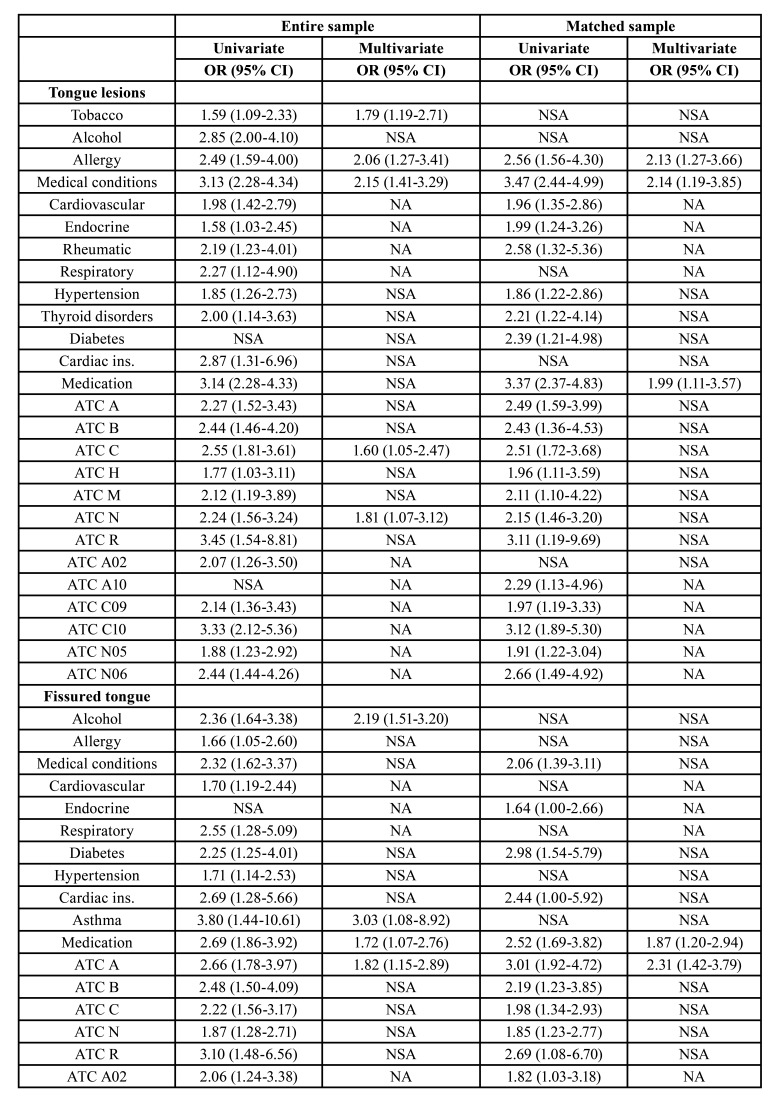
Statistically significant associations (*p*<0.05) according to the logistic regression and the propensity score-matched (PSM) analyses.

**Table 4 cont.**
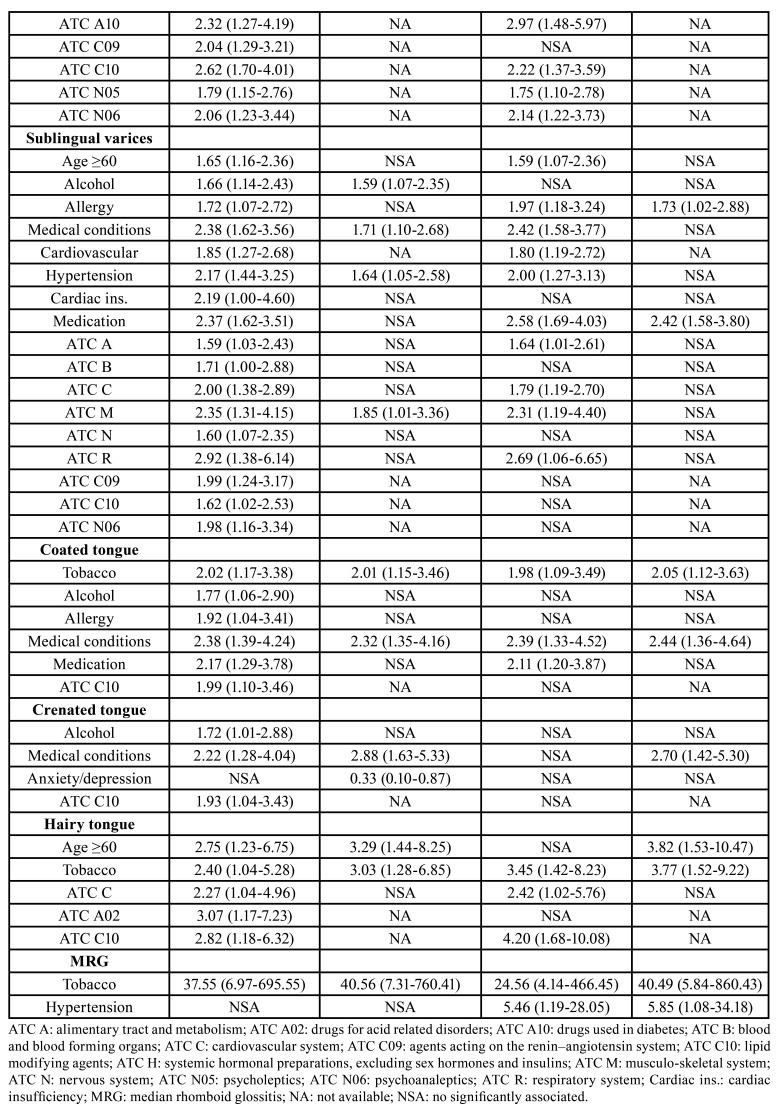
Statistically significant associations (*p*<0.05) according to the logistic regression and the propensity score-matched (PSM) analyses.

## Discussion

This case-control study showed that patients with tongue lesions were more likely to suffer from allergies (OR 2.13) or medical conditions (OR 2.14), and more likely to be taking medication (OR 1.99). By adjusting age, gender, tobacco use, and alcohol use through PSM to minimize selection bias, we assessed the impact of medical conditions and medications on different lingual pathologies.

Many studies have documented gender differences in relation to the frequency of tongue lesions. Some authors have reported a predominance of fissured tongue (3,4,10,11,22) and hairy tongue in men (3,5,10,11), while others have noted a predominance of crenated tongue (11) and even fissured tongue in women (17). We confirmed that, as some authors have previously reported (2,13,14,20), gender is not relevant in the occurrence of tongue lesions.

Age was only associated with hairy tongue, yielding an OR very similar to that reported by Mumcu *et al*. (OR 3.29 and OR 3.5, respectively) (2). In contrast, our results did not support a relationship with sublingual varices (2,6-8) or fissured tongue (5,22). In our study, smoking was related to both coated (3,11) and hairy tongue (3,10). The OR for coated tongue (OR 2.05) differed from the OR reported by Campisi & Margiotta (OR 8.08), which was adjusted for age (23). Our OR for hairy tongue (OR 3.77), was lower than the OR produced by the multivariate model from Tortorici *et al*. for coated/hairy tongue (OR 4.8) (18), and it was much lower than the OR reported by Mumcu *et al*. (OR 9.3) (2). Although coated and hairy tongue are considered to have a multifactorial aetiology (24), tobacco may be a risk factor for these lesions, but once adjusted for other variables, its impact is lower than reported in previous studies.

Tobacco was strongly associated with MRG. This association has already been suggested. Arendorf & Walker observed that 85% of MRG patients were smokers (25). Mehta *et al*. noted that MRG regresses in a higher proportion among those individuals who quit the habit (26). Additionally, Pentenero *et al*. observed that alcohol consumption increases the risk of MRG (OR 2.69) (27), although we did not corroborate this in our study. Although alcohol was associated with fissured tongue and sublingual varices in our multivariate analysis, these associations disappeared in the PSM analyses.

The use of dentures was inversely associated with crenated tongue, but in the multivariate PSM model this association was ruled out. A relationship between dentures and sublingual varices had been shown in two studies (6,7), however, like Mumcu *et al*. (2), we were unable to corroborate it.

The history of allergies may be a risk factor for sublingual varices (OR 1.73). To date, no other study has reported this relationship. In addition, we confirmed the lack of association between allergy and geographic tongue, in line with Shulman & Carpenter (14), but contrary to reports from other authors (13,28).

Despite the associations noted between tongue lesions and medical conditions or medications in our univariate analysis, they were not as important in the multivariate PSM model. Nevertheless, medical conditions may be a risk factor for coated tongue (OR 2.44) and crenated tongue (OR 2.70). The association between chronic diseases and lingual pathology was described in a study in children which gave an OR very similar to ours (OR 2.23 vs OR 2.14) (15).

In the multivariate PSM model, arterial hypertension was the only disease that was associated with a tongue lesion. To the best of our knowledge, this is the first study to establish this association with MRG. In the univariate analysis, fissured tongue was the lesion that was most frequently associated with medical conditions, such as cardiovascular and respiratory diseases, hypertension, cardiac insufficiency, asthma, and diabetes, as previously described by Koay *et al*. (4). However, we were unable to confirm any of these associations in the final PSM model.

The relationship between sublingual varices and cardiovascular diseases in the univariate analysis and with hypertension in the multivariate model on the full sample before PSM is in line with data from other authors (6-8,17). It has been suggested that an increased venous pressure may play a role in the development of sublingual varices (8,17). Nonetheless, the absence of corroboration in the PSM indicates the possible confounding factors present in previous studies.

In the study by Dafar *et al*. (12), geographic tongue was associated with hypertension (OR 1.7). However, we could not establish an association between geographic tongue and diabetes, psychological disorders, or other medical conditions, which is in line with Shulman & Carpenter and Miloğlu *et al*. (13,14).

In our study, anxiety/depression was inversely associated with crenated tongue in the multivariate model before the application of PSM. This tongue lesion is considered as a manifestation of bruxism (29), and a clinical sign of stress (30) but since parafunctional habits and psychological profile of stress have not been registered in our study, and they may have acted as possible confounders.

Medication intake constituted a possible risk factor for sublingual varices (OR 2.42) and fissured tongue (OR 1.87). Agents acting on the alimentary tract and metabolism were also related to fissured tongue (OR 2.31). Lynge Pedersen *et al*., studied a sample of older Danish people and found that sublingual varices were also more prevalent in patients taking daily medication (17). In addition, Jahanbani *et al*. (5), in an Iranian population, found that in patients with fissured tongue the risk of taking some kind of medication was doubled when compared to those without this lesion, after adjusting for age and gender. In this sense, the hyposalivation effect of certain medications may contribute to the development of tongue pathology. In the study by Lynge Pedersen *et al*. (17) fissured tongue was more frequent in individuals taking daily medication than in non-medicated (10.2% vs 4.7%), and this lesion was associated with low unstimulated whole saliva flow rates and xerostomia. Nevertheless, there is limited data regarding the effect of medication and hyposalivation on lingual pathology.

A strength of our study is the large number of patients with fissured tongue and sublingual varices, which is very similar to other epidemiological studies (1,2,17). However, it has some limitations. One limitation is the small number of certain lesions, such as MRG. Another is the wide range of the confidence interval for smoking in relation to MRG. Lastly, although PSM reduces the sample size, it reduces bias, reinforcing the quality of the analysis.

In conclusion, as far as we are aware, this is the first study on lingual pathology that includes a PSM analysis. The results suggest that a history of allergies, the presence of medical conditions, and the use of medication are associated with increased probability of tongue lesions. Smoking may be a risk factor for coated tongue, hairy tongue and MRG. The analysis of diseases and medications by subgroups requires studies matched by habits with larger sample sizes, in order to corroborate our observations.
